# Limits of Memory-Assisted Coherence in Cavity-Embedded Molecules

**DOI:** 10.1021/acs.jpclett.6c02396

**Published:** 2026-10-05

**Authors:** Joshua C. Kinslow, Ben S. Humphries, Dale Green, Magnus O. Borgh, Garth A. Jones

**Affiliations:** † School of Chemistry, Pharmacy and Pharmacology, 6106 University of East Anglia, Norwich Research Park, Norwich NR4 7TJ, United Kingdom; ‡ Department of Physics and Astronomy and Thomas Young Centre, University College London, Gower Street, London WC1E 6BT, United Kingdom; § School of Engineering, Mathematics and Physics, 14295 University of East Anglia, Norwich Research Park, Norwich NR4 7TJ, United Kingdom

## Abstract

Strong light–matter coupling in optical cavities is being widely explored as a strategy for modifying molecular dynamics and enhancing coherence. At the same time, structured vibrational environments can exhibit non-Markovian memory that promotes information backflow and coherence revival. We investigate the competition between these effects in a vibronic molecular system strongly coupled to a lossy optical cavity. Using hierarchical equations of motion to treat structured vibrational baths, we quantify both system coherence and non-Markovianity via the *l*_1_ norm of coherence and the Breuer–Laine–Piilo measure, respectively. We find that cavity coupling enhances coherence in overdamped environments but can suppress memory-assisted coherence in resonant underdamped regimes, even in the absence of significant photon loss. This suppression arises from hybridization-induced modification of system–bath information flow. We demonstrate that cavity coupling does not universally protect coherence but establishes limits governed by the competition between vibrational recurrence time scales and polaritonic hybridization.

## Graphical abstract

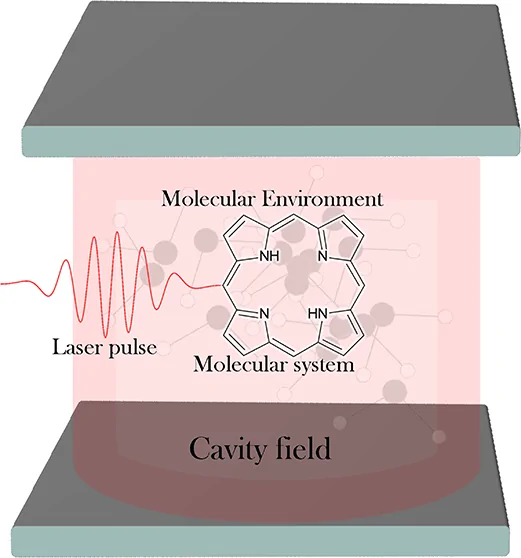

Strong light–matter coupling in optical cavities has emerged as a powerful strategy for modifying molecular dynamics, enabling control over spectroscopy,^1^ energy transfer,^2^ and chemical reactivity beyond what is achievable in solution.^3^ Recent work has focused on understanding the dynamical mechanisms underlying cavity-modified chemistry,^4,5^ particularly in the context of polaritons, which evolve during hybridization with confined cavity fields.^6–12^ At the same time, advances in open-quantum-systems theory have highlighted environmental memory (non-Markovianity) as a potential resource^13,14^ for preserving coherence in complex vibronic systems, where structured vibrational environments can support information recurrence and coherence revival.^15–17^ However, non-Markovianity can also accelerate decoherence when the bath returns information out of phase with the system’s evolution.^18,19^

Previous studies have established how light–matter coupling modifies open system dynamics, with Canaguier-Durand et al.^20^ and del Pino et al.^21^ identifying non-Markovian relaxation and collective nuclear effects in organic polaritons, while Takahashi and Watanabe^22^ showed how hybridization allows for decoupling from a thermal bath. Building on this, Rouse et al.^23^ demonstrated that strong molecular vibrations drive multi-phonon dephasing and shift polariton energies, complementing broader demonstrations of cavity-extended electronic coherence by Hu et al.^24^ These parallel developments therefore starkly raise the central and unresolved question: how does cavity-induced dissipation interact with memory-assisted coherence in molecular systems?

In cavity strong-coupling platforms, molecular ensembles reside in structured vibrational environments while photon leakage through imperfect mirrors introduces an additional loss channel. In this work, we investigate how coherence dynamics are governed by a competition between the spectral recurrence of the vibrational bath and the dynamic restructuring of the system’s energy landscape through light–matter hybridization.^25–27^ When hybridization shifts the system’s eigenstructure, it may disrupt the resonance required for memory-assisted coherence through non-Markovian recurrence.

Determining how this competition unfolds is essential for understanding when cavity coupling protects coherence and when it undermines environmentally assisted quantum dynamics. Here, we model a vibrational system embedded within a strongly coupled cavity, with multiple environmental structures and parameters,^28^ using the hierarchical equations of motion and Lindblad dynamics. By measuring system coherence as well as the degree of non-Markovianity,^16,29^ we look at the interplay of the system, cavity, and environment to understand how information is exchanged between them.

We model the system as an open quantum vibronic molecule with two electronic states separated by ω_eg_ = 16 000 cm^–1^, each coupled to an intramolecular vibration of frequency ω_0_ = 350 cm^–1^ with dimensionless displacement Δ = 0.8. The vibrational coordinate interacts with a thermal environment represented by a continuum of harmonic oscillators, with either an overdamped or underdamped spectral density to tune the degree of environmental memory. In the underdamped case, the principal peak of the spectral density is centered at 350 cm^–1^ resonant with the molecular vibrational mode.

The molecule is strongly coupled to a single quantized cavity mode, resonant with the electronic transition, ω_c_ = ω_eg_, producing polaritonic states under light–matter hybridization, while photon leakage introduces an additional dissipative channel. The cavity quality factor is *Q*_cav_ = ω_c_/κ, where κ is the cavity decay rate.^30^ We consider a near-lossless cavity allowing us to isolate hybridization effects from irreversible photon loss. The light–matter coupling strength is set to *g* = 1000 cm^–1^, putting us firmly in the strong coupling regime, where *g* is much greater than both κ and the relaxation rate of the molecule.^8,31–33^

This coupling strength, representing the single-molecule, strong-coupling regime achievable in plasmonic nano- or picocavities, was selected to clearly expose the competition between polaritonic restructuring and environmental recurrence.

The total system is described by a Hamiltonian comprised of molecular (*Ĥ*_mol_),^34,35^ cavity (*Ĥ*_cav_),^36,37^ environmental (*Ĥ*_bath_),^28^ interaction (*Ĥ*_mol–cav_ and *Ĥ*_mol–bath_),^25,38^ and field (*Ĥ*_laser_)^16^ contributions

$${Ĥ}_{\mathrm{tot}}={Ĥ}_{\mathrm{mol}}+{Ĥ}_{\mathrm{cav}}+{Ĥ}_{\mathrm{mol}-\mathrm{cav}}+{Ĥ}_{\mathrm{bath}}+{Ĥ}_{\mathrm{mol}-\mathrm{bath}}+{Ĥ}_{\mathrm{laser}}.$$ 1

The vibrational degrees of freedom are coupled to spectral densities that range from near-Markovian to strongly non-Markovian regimes. This bath captures reversible intramolecular vibrational relaxation and dephasing. In contrast, cavity losses arise from photon leakage through imperfect mirrors and are well-described by a Markovian dissipator acting on the cavity mode. Treating the bath and cavity with independent operators reflects their distinct physical time scales and avoids introducing spurious correlations. Simulations are initiated by excitation with a Gaussian laser pulse, using a semi-classical approximation of a laser field.^39,40^ The laser frequency (16 000 cm^–1^) satisfies the resonance condition between the molecule and the cavity, thus allowing us to investigate how the resulting hybridization restructures the energy landscape in real time.

We first employ the underdamped spectral density, where certain bath modes will have a much stronger coupling, with less damping. This structured spectral density is given as^16,41^

2 $$J\left(\omega \right)=\frac{2\eta \gamma {\omega_{0}}^{2}\omega }{{\left({\omega_{0}}^{2}-\omega^{2}\right)}^{2}+\gamma^{2}\omega^{2}}$$

which includes a peak at ω_0_. Broadening of this peak depends on the damping parameter, γ. In the limit ω_0_ ≪ γ, the bath becomes overdamped and eq 2 reduces to the Lorentz–Drude form

3 $$J\left(\omega \right)=2\eta \frac{\omega \Lambda }{\omega^{2}+\Lambda^{2}}$$

with bath reorganization energy η and dissipation rate Λ. This spectral density couples at lower frequency modes, with a peak at ω = Λ.

We treat overdamped and underdamped spectral densities as distinct limiting regimes to deconstruct how light–matter hybridization interacts with different environmental structures. In the overdamped case, reducing Λ shifts the broad spectral weight toward lower frequencies, whereas in the underdamped case, reducing γ narrows the Lorentzian line width around resonance ω_0_ while conserving the bath reorganization energy of η = 30 cm^–1^. While both parameter variations alter the environmental correlation time (τ_c_), the underdamped bath establishes a highly frequency-selective, non-Markovian interaction channel.

For the dynamics, we employ the hierarchical equations of motion (HEOM),^29,42–46^ which provide a numerically exact description of system–bath dynamics for harmonic environments across varying damping regimes: this accurately captures the memory effects of the environment. Cavity dissipation is incorporated through Lindblad operators^47^ acting on the cavity field, enabling the inclusion of irreversible photon loss. HEOM–Lindblad formalisms have been used for other polariton studies.^48–50^ Full details of our model can be found in the Supporting Information.

All simulations are initialized from thermal equilibrium, with the entire population in the electronic ground state. The system is then propagated following excitation by a Gaussian laser pulse tuned to ω_eg_. Both coherence and information flux are calculated after excitation, tracking the system dynamics over 2 ps. Full details of the Hamiltonian, bath parametrization, hierarchy construction, and numerical convergence are provided in the Supporting Information.

Coherence dynamics are quantified using the *l*_1_ norm of coherence, defined as the sum of the magnitudes of the off-diagonal elements of the reduced density matrix in the full molecular basis^51–56^

$$C_{l_{1}}\left(\rho \right)=\underset{i\neq j}{\sum }\left|\rho_{ij}\right|.$$ 4

This measure provides a direct indicator of quantum superposition and is well-suited for tracking decoherence in complex molecular systems. While the *l*_1_ norm of coherence is basis-dependent, we evaluate the *l*_1_ norm over the full joint molecular cavity density matrix ρ in the fixed bare vibronic basis, as this total polaritonic state space provides a physically intuitive representation of the combined light–matter degrees of freedom probed in ultrafast spectroscopy. We extract the rate of decoherence from the slope of these curves, where a steeper negative gradient indicates accelerated decoherence.

To characterize environmental memory, we employ the Breuer–Laine–Piilo (BLP) measure of non-Markovianity^15,52,57–60^

$$N\left(t\right)=\underset{Ḋ\left(t^{\prime }\right)>0}{\int_{0}^{t}}\frac{d}{dt^{\prime }}D\left(\rho_{1}\left(t^{\prime }\right),\rho_{2}\left(t^{\prime }\right)\right)dt^{\prime }.$$ 5

Here, *D* is the trace distance of the system, and *Ḋ* is the full time derivative, measuring the distinguishability between the two states with and without excitation, ρ_1_ and ρ_2_. This diagnostic quantifies information backflow by monitoring the time evolution of the distinguishability between two system states. We interpret N as a probe of the information backflow rather than a general measure of the total available environmental memory. The BLP measure is evaluated here for a specific distinguishability protocol, comparing the system evolution with and without laser excitation. Therefore, both trajectories undergo identical equilibration procedures, thereby establishing the same equilibrium system–bath preparation. Trace-distance measures following such correlated preparations have been considered previously,^16,61^ and initially correlated open-system dynamics can more generally be represented by a set of completely positive maps.^62^ After the laser field terminates, both systems undergo free evolution.

For our simulations, we choose parameter regimes that highlight the competition between vibrational bath recurrence and cavity photon loss, enabling direct assessment of the limits of memory-assisted coherence in molecular cavity quantum electrodynamics (QED). All simulations are run at 298 K, with measurements taken after the system is excited by a laser pulse tuned to ω_eg_ with an intensity of χ = 10^11^ V m^–1^.^16^ Tuning the laser to this frequency ensures that we are probing the system at the exact point where the light and matter are most strongly coupled. Further details are given in the Supporting Information.

To correlate the change in coherence with the degree of non-Markovianity, we consider the BLP measure for a series of overdamped and underdamped baths by changing Λ (bath dissipation rate) and γ (bath damping strength), respectively. We perform simulations both with and without cavity coupling for all baths, to establish how the cavity modifies the net information flux. The BLP is evaluated on the reduced density matrix of the electronic, vibrational, and cavity degrees of freedom collectively (after tracing out the baths). In the left column of Figure 1, we consider a molecule without cavity coupling but coupled to overdamped (Figure 1A) and underdamped (Figure 1C) baths. In the overdamped case, for the fastest rate (140 cm^–1^), there is near-Markovian dynamics; when it is slowest (40 cm^–1^), non-Markovianity is at its highest, with moderate backflow of information from the bath to the system. In the case of the underdamped bath (Figure 1C), we also see an increasing N with a decreasing γ: information backflow is an order of magnitude higher for the underdamped case compared to the overdamped case, across all three bath relaxation rates.

**Figure 1:**
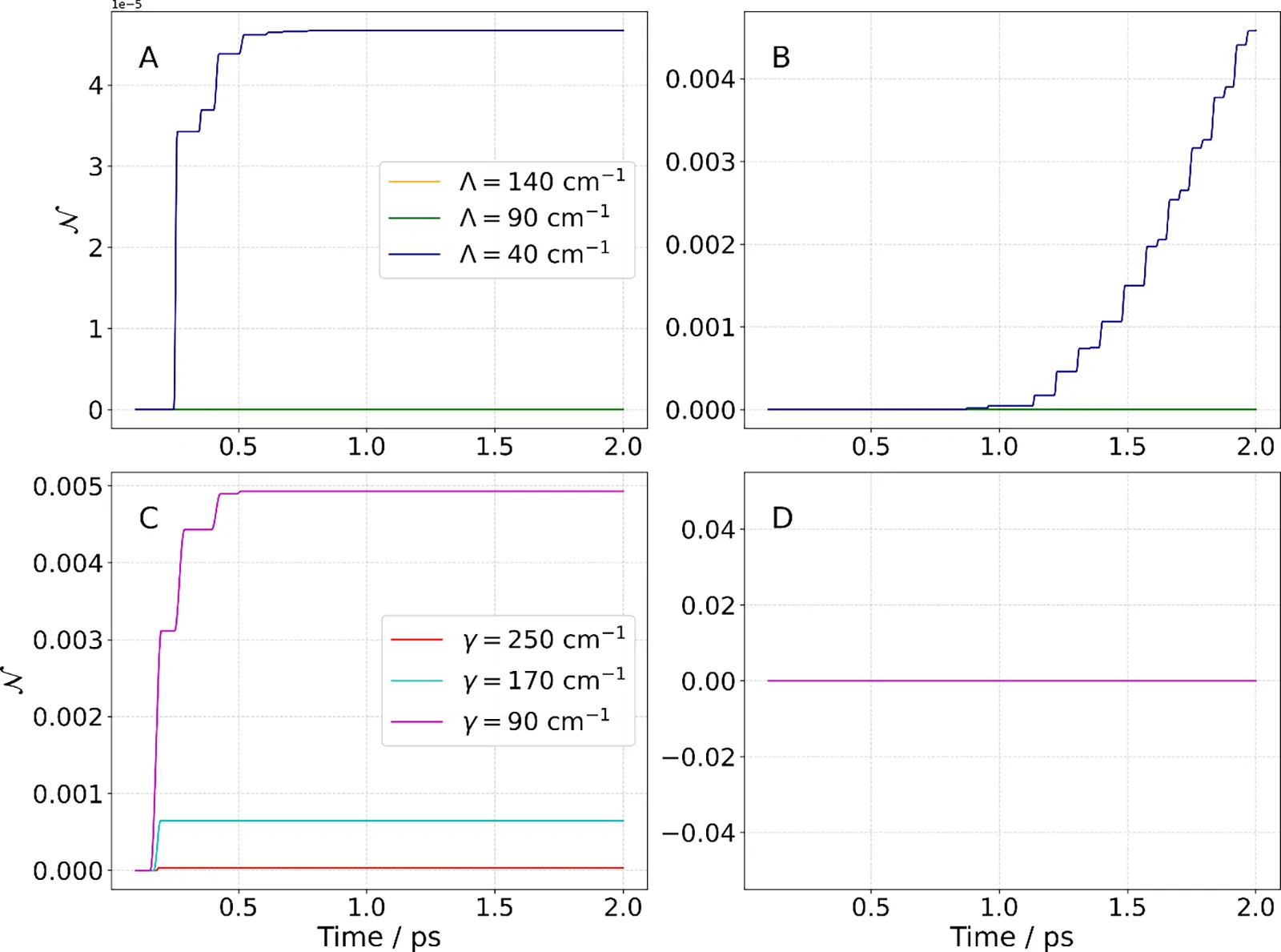
Accumulated BLP measure for the full system (ρ) without cavity coupling is shown in panels A and C. Panel A is coupled to a bath with an overdamped spectral density, across three bath recovery rates (Λ = 140, 90, and 40 cm^–1^). Λ = 140 is overlapped by the Λ = 90 result. Panel C shows our system coupled to an underdamped spectral density also with three bath recovery rates (γ = 250, 170, and 90 cm^–1^). On the right, we show the BLP of the system with strong cavity coupling of *g* = 1000 cm^–1^ (panels B and D, respectively). Note, to ensure a consistent comparison between the coupled and uncoupled regimes, our cavity-free baseline results are calculated using the same total Hilbert space as the coupled cases but with the light–matter coupling set to *g* = 0 cm^–1^. This ensures that the BLP measure is evaluated on a system of identical dimensionality, confirming that the observed results are a dynamical consequence of hybridization rather than an artifact of the state space definition.

The right column of Figure 1 shows the effect of strong cavity coupling on the effective non-Markovianity of the molecule–cavity subsystem. In the overdamped case (Figures 1B), cavity coupling increases the accumulated BLP measure, indicating enhanced information backflow. In contrast, for the underdamped bath (Figure 1D), the introduction of strong light–matter coupling causes a decrease of the accumulated BLP signal, which becomes nearly zero across the explored bath parameters.

Importantly, this reduction in BLP is not solely attributable to photon leakage. To isolate the role of hybridization, we consider the limit of vanishing cavity dissipation (κ = 0). Even in this lossless limit, the accumulated BLP measure remains reduced relative to the uncoupled case, with $N\left(t=2\mathrm{ps}\right)=8.29\times 10^{-4}$ when γ = 90 cm^–1^ (see the Supporting Information for details). This demonstrates that hybridization-induced detuning decreases the influence of environmental recurrence, even in the limit of zero photon leakage.

Strong coupling reshapes the system eigenstructure through polaritonic splitting,^5,27,63^ modifying the frequencies where the system–bath interactions are strongest and altering the phase relationship between bath recurrence and system dynamics. In the underdamped regime, where the bath is resonant with the cavity-uncoupled vibrational mode, hybridization detunes this by shifting the vibrational mode, ensuring that constructive information backflow is reduced, even in the absence of additional dissipative channels. The cavity therefore modifies system–bath information flow not only by introducing loss but also by fundamentally restructuring the coherent dynamics of the coupled subsystem.

To investigate the decoherence of our system, we track its rate by extracting the slope of log­(*C*_*l*_1__) and take the partial traces over the separate subsystems to isolate their individual coherence contributions, defining ρ_mol_ = Tr_cav_(ρ), ρ_vib_ = Tr_cav,elec_(ρ), and ρ_elec_ = Tr_cav,vib_(ρ). We first consider the overdamped case in the top panels of Figure 2, with Λ = 90 cm^–1^. Without the presence of the cavity (Figure 2A), the rate of decoherence of the vibrational degrees of freedom (green) is significantly greater than purely electronic decoherence (blue). On the other hand, when coupled to the cavity, the vibrational decoherence, shown in panel B of Figure 2, has slowed and is more similar to that of the electronic coherence (Figure 2B). With cavity coupling, we observe oscillations in log­(*C*_*l*_1__) of both ρ_mol_ and ρ_vib_ at 2000 cm^–1^. This is a feature of the light–matter hybridization, where Rabi flopping occurs between low and high polariton states, split by 2*g*.

**Figure 2:**
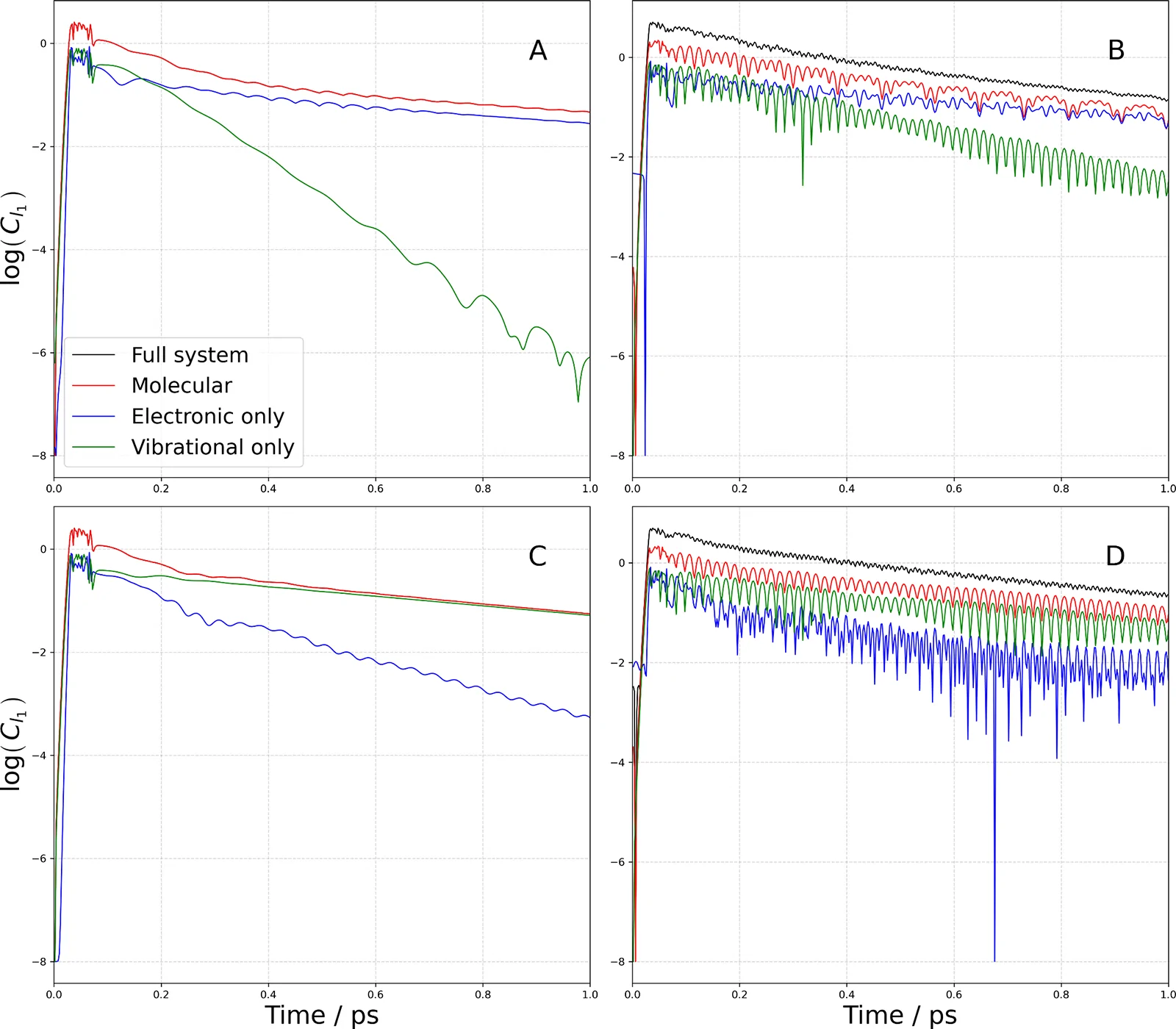
Log­(*C*_*l*_1__) for ρ (black line), ρ_mol_ = Tr_cav_(ρ) (red line), ρ_vib_ = Tr_cav,elec_(ρ) (green line), and ρ_elec_ = Tr_cav,vib_(ρ) (blue line) over 1 ps. Panel A shows the system coupled to an overdamped bath with Λ = 90 cm^–1^ without cavity coupling, and panel B shows it with strong cavity coupling (*g* = 1000 cm^–1^). Panel C shows the system coupled to the underdamped bath, γ = 170 cm^–1^, without cavity coupling, and panel D shows it with strong cavity coupling. Due to the lack of coupling between the molecule and the cavity, panels A and C have identical results when tracing out the cavity states of the system (red and black lines).

In the case of the underdamped bath (Figure 2C), with a central frequency of 350 cm^–1^ and a damping rate of γ = 170 cm^–1^, we see a qualitatively different characterization of the decoherence. The rate of electronic decoherence is greater than that of the vibrational decoherence and is nearly identical to the rate of decoherence of the total system. The highly structured environment suppresses vibrational decoherence. With the addition of the cavity (Figure 2D), the electronic decoherence has changed following light–matter hybridization, yet it is not as significant of an improvement as with the vibrational coherence in the overdamped case.

To map how environmental time scales alter these dynamics, we track the total system *l*_1_ norm while systematically varying the bath parameters, Λ for the overdamped spectral density and γ for the underdamped, to assess the impact of these parameters on the decoherence dynamics without and with cavity coupling (left and right, respectively) in Figure 3. By using the total system as our diagnostic baseline, we ensure that the reported dynamics capture the full phase evolution of the hybridized manifold as it interacts with the vibrational environment.

**Figure 3:**
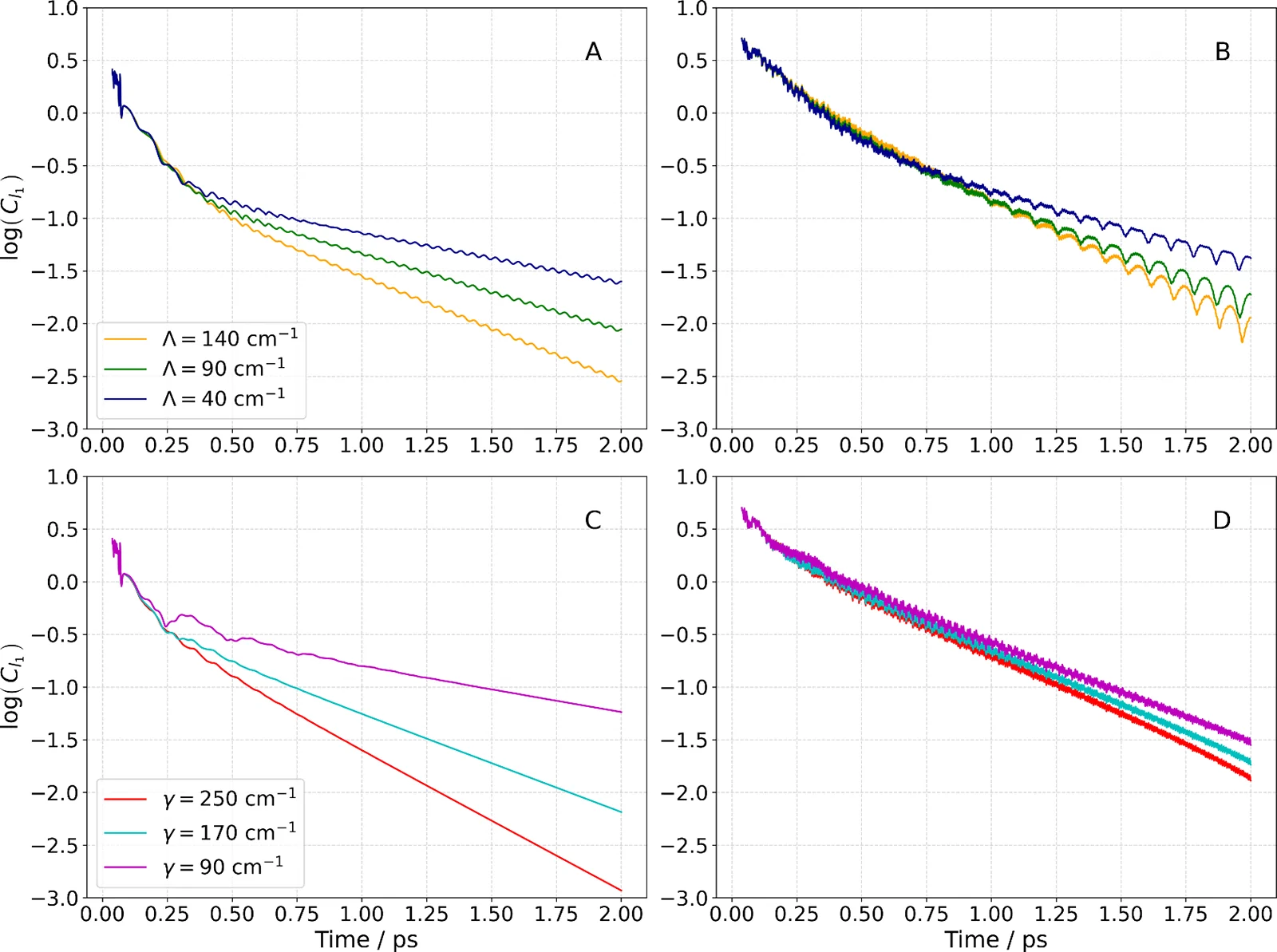
We show the rate of decoherence, log­[*C*_*l*_1__(ρ)], for the total polaritonic coherence, coupled to an (A and B) overdamped bath or (C and D) underdamped bath. In panels A and C, the molecule is uncoupled from the cavity (*g* = 0 cm^–1^). In panels B and D, the molecule is strongly coupled to the cavity *g* = 1000 cm^–1^. We show the rates for three different bath relaxation rates over 2 ps.

For the overdamped results (Figure 3A and B), we can see a clear decrease in the rate of decoherence when the cavity is coupled strongly to the system (Figure 3B) compared to uncoupled (Figure 3A). In the case of no cavity coupling, the reduction of the relaxation speed of the bath Λ (from 140 to 40 cm^–1^), corresponding to increased non-Markovianity, decreases the rate of decoherence.

When the system is coupled to an underdamped bath (bottom panels), the addition of the cavity decreases the rate of decoherence for the two fastest bath decay rates (γ = 250 and 170 cm^–1^). However, for the slowest (γ = 90 cm^–1^), the environment has greater assisted coherence, which disrupts this trend. Without the cavity (*g* = 0 cm^–1^), the vibrational transition is perfectly resonant with this structured bath, allowing the system to benefit from a highly persistent, in-phase information backflow that maintains coherence over longer time scales.

Finally, to further elucidate the competition between polaritonic restructuring and environmental non-Markovian effects, we performed a systematic sweep of the light–matter coupling strength (*g*). As shown in Figure 4A, introducing a weak cavity coupling (*g* = 20–100 cm^–1^) interrupts this mechanism. In this range, the underlying eigenvalues of the hybridized system matrix begin to diverge, which shifts the internal frequencies away from the 350 cm^–1^ resonance. Because the timing of the environmental recurrence is driven out of phase with the hybridized dynamics, the non-Markovian recurrence interferes destructively, with the maximum decoherence rate at *g* = 100 cm^–1^. As illustrated in Figure 4B, the increasing of the coupling strength moves the system into a polaritonic shielding regime. The electronic splitting drives the polaritonic branches out of the underdamped bath’s Lorentzian peak, decoupling the hybrid system from the local environmental noise. For γ = 250 and 170 cm^–1^, this protective shielding supersedes the loss of non-Markovian effects; therefore, the coherence improves compared to the uncoupled baseline.

**Figure 4:**
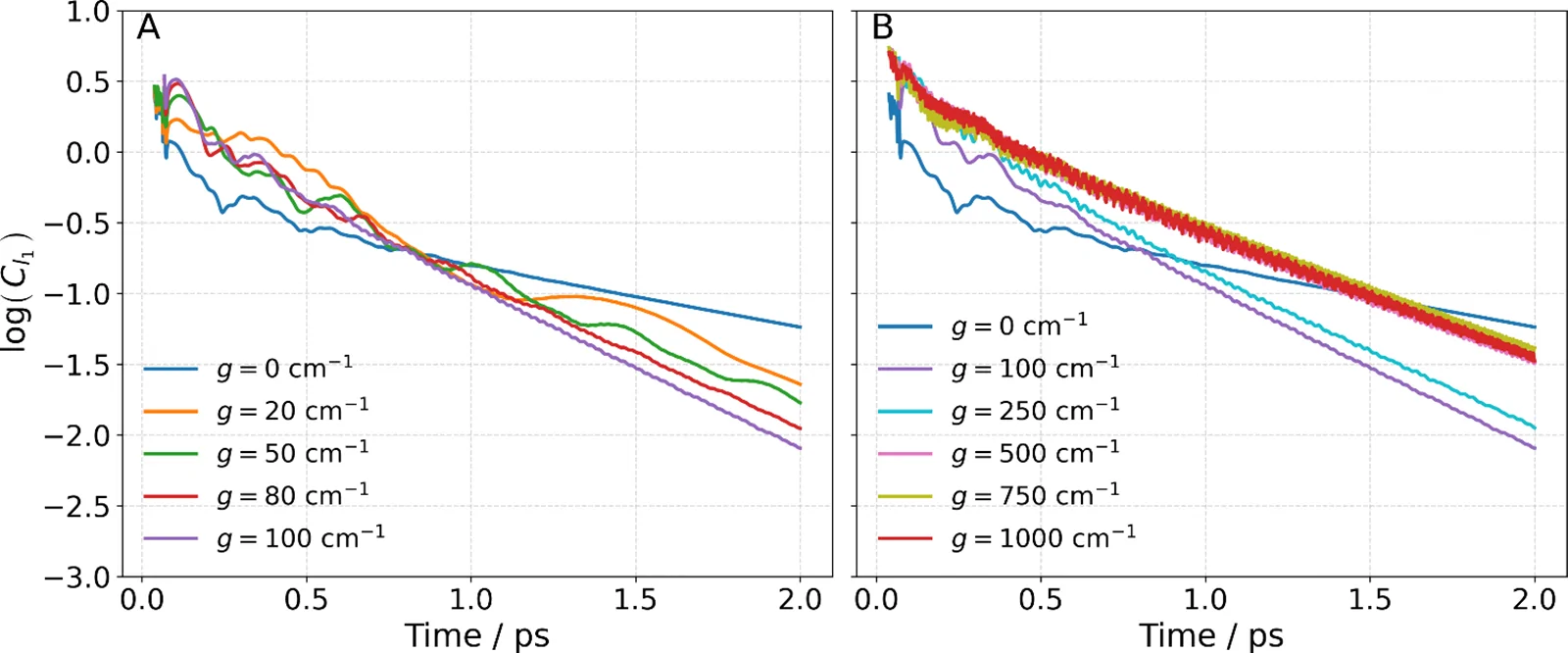
Systematic exploration of the light–matter coupling strength (*g*) on the *l*_1_ norm of the total polaritonic coherence for the system coupled to an underdamped bath (γ = 90 cm^–1^). The *g* = 0 cm^–1^ baseline represents the uncoupled molecule. Panel A demonstrates that weak coupling (*g* = 20–100 cm^–1^) induces a resonance mismatch that accelerates decoherence. Panel B shows that, at stronger coupling (*g* ≥ 500 cm^–1^), the polaritonic states detune from the resonant peak, leading to the observed saturation and recovery of the coherence rate.

We have investigated the interplay between non-Markovian recurrence from the vibrational environmental and cavity photon loss in a molecular system strongly coupled to a cavity. Our findings for the overdamped regime are consistent with previous reports of cavity-enhanced coherence, which demonstrate that light–matter hybridization can slow decoherence in molecular systems by decoupling the system from an environment.^24,64^ In such cases, the cavity acts as a protective shield, detuning it from the broad spectral density of the bath to extend polaritonic lifetimes. By systematically varying the bath structure and cavity strength, we identify a clear competition between bath-mediated information backflow and light–matter hybridization. In overdamped environments, strong cavity coupling enhances coherence and increases the effective memory accessible to the molecule–cavity subsystem. However, in structured underdamped environments resonant with molecular vibrations, we observe a qualitatively different behavior: cavity coupling can suppress non-Markovian information backflow and increase decoherence rates due to hybridization disrupting the resonance between the system and its environment, even when photon loss is minimal.

While this study focuses on the single-molecule limit, representative of the extreme field confinement found in plasmonic nanocavities,^65,66^ it provides a fundamental basis for understanding dynamics in collective strong-coupling regimes. In an ensemble of *N* molecules, the bright polariton states experience a collective Rabi splitting that scales as √*N*,^67,68^ detuning them even further from the local vibrational resonances identified here. Consequently, the suppression of environmentally assisted coherence is expected to persist and potentially intensify for the bright manifold. In contrast, the *N* – 1 nearly degenerate dark states remain resonant with the bare molecular transition and are thus expected to continue sampling the resonant bath peak, maintaining their environmentally assisted coherence.^25^ This suggests that, in collective systems, the interplay between environmental non-Markovian effects and cavity coupling is state-selective, offering a potential mechanism for the targeted control of decoherence in polaritonic ensembles.^64^

These results demonstrate that cavity coupling does not generically protect quantum coherence. Rather, it reshapes system–bath information flow and, in highly non-Markovian regimes, can interfere with the very memory effects that sustain coherence. The degree of coherence enhancement is therefore governed by a competition between vibrational recurrence and polaritonic hybridization dynamics. Our findings establish practical limits on memory-assisted coherence in molecular cavity QED and provide guidance for identifying regimes in which strong light–matter coupling enhances or instead suppresses environmentally mediated quantum protection of molecular systems.

## Supplementary Material



## Notes

Full details of the Hamiltonian model, theoretical details of the HEOM–Lindblad master equation, and tabular BLP values ( N ) for lossless (κ = 0) and lossy cavity regimes (PDF)
