# Supplementary material for: Limits of Memory-Assisted Coherence in Cavity-Embedded Molecules
Source: J Phys Chem Lett. 2026 Oct 5;17(41):11723–30. doi: 10.1021/acs.jpclett.6c02396 (PMC13679082; doi:10.1021/acs.jpclett.6c02396)
Supplement: Supplementary file 1 [file jz6c02396_si_001.pdf]

Limits of Memory-Assisted Coherence in Cavity Embedded Molecules - Supporting Information

Joshua C. Kinslow,† Ben S. Humphries,‡ Dale Green,¶ Magnus O. Borgh,¶ and Garth A. Jones*,†

†*School of Chemistry, Pharmacy and Pharmacology, University of East Anglia, Norwich Research Park, Norwich, NR4 7TJ, UK*

‡*Department of Physics and Astronomy and Thomas Young Centre, University College London, Gower Street, London, WC1E 6BT, UK*

¶*School of Engineering, Mathematics and Physics, University of East Anglia, Norwich Research Park, Norwich, NR4 7TJ, UK*

E-mail: garth.jones@uea.ac.uk

1 Hamiltonian

Our Hamiltonian describes the full model, with two separate environments and coupling operators for both the molecule coupled environment and cavity coupled environment, which evolve independently. The environments are represented by an ensemble of infinite quantum harmonic oscillator modes. Our total Hamiltonian is described as

$$\hat{H}_{\text{Tot}} = \hat{H}_{\text{mol}} + \hat{H}_{\text{cav}} + \hat{H}_{\text{mol-cav}} + \hat{H}_{\text{bath}} + \hat{H}_{\text{mol-bath}} + \hat{H}_{\text{laser}}, \tag{1}$$

with $\hat{H}_{\text{mol-bath}}$ being the coupling between the molecule and the vibronic bath and $\hat{H}_{\text{mol-cav}}$ describing the coupling between the molecule and the optical cavity . The vibrational degrees of freedom for our molecule are described by

$$\hat{H}_{\text{mol}} = |g\rangle \, \hat{h}_g \, \langle g| + |e\rangle \, \hat{h}_e \, \langle e| \,, \tag{2}$$

where the nuclear Hamiltonian for the electronic ground state is

$$\hat{h}_g = \hbar\omega_0(\hat{b}^\dagger\hat{b} + \tfrac{1}{2}), \tag{3}$$

while that of the excited state is given by

$$\hat{h}_e = \hbar(\omega_{eg} + \lambda) + \hbar\omega_0 \left[\hat{b}^\dagger\hat{b} - \sqrt{\frac{\lambda}{\omega_0}} \left(\hat{b} + \hat{b}^\dagger \right) + \frac{1}{2} \right]. \tag{4}$$

Vibrational relaxation occurs as phonons leave the system and escape to the environment. The environment is modelled as a series of infinite harmonic oscillators, with a distribution of coupling strengths determined by a spectral density. We describe the coupling between the molecule and the bath as

$$\hat{H}_{\text{bath}} + \hat{H}_{\text{mol-bath}} = \sum_a \frac{\hat{p}_\alpha^2}{2m_\alpha} + \frac{1}{2} m_\alpha \omega_\alpha^2 \Big(\hat{x}_\alpha - \frac{g_\alpha \hat{Q}}{2m_\alpha\omega_\alpha^2} \Big)^2 \tag{5}$$

$\hat{Q} = (\hat{b} + \hat{b}^\dagger)/\sqrt{2}$. m_α, $\hat{p}_\alpha$ and $\hat{x}_\alpha$ are the mass, momentum and coordinate of the harmonic modes of the environment. The coupling strength, at the αth harmonic oscillator, is determined by the spectral density, with a bath reorganisation energy of $\eta = 30$ cm^{-1}.

We also describe the relationship between the electronic levels of the molecule and the strongly coupled optical cavity. The Hamiltonian for the cavity field is given as

$$\hat{H}_{\text{cav}} = \hbar\omega_c \hat{a}^\dagger \hat{a}, \tag{6}$$

where the $\hat{a}^\dagger$ and $\hat{a}$ are the bosonic creation and annihilation operators, with ω_c representing the cavity mode frequency. The interaction Hamiltonian, $\hat{H}_{\text{mol-cav}}$, describes the relationship between the cavity field and the electronic degrees of freedom of the molecule. It is given as

$$\hat{H}_{\text{mol-cav}} = \frac{\hbar\Omega}{2}\hat{E}\hat{S}, \tag{7}$$

where Ω is the Rabi frequency, which is characterised by the strength of the light-matter interaction. The molecular polarization operator is defined as $\hat{S} = \hat{\sigma}_+ + \hat{\sigma}_-$, where $\hat{\sigma}_+ = |e\rangle\langle g|$ and $\hat{\sigma}_- = |g\rangle\langle e|$ are the electronic raising and lowering operators of the molecule, respectively. The quantized electric field operator is given by $\hat{E} = (\hat{a} + \hat{a}^\dagger)$. To take the measurements required of this system to determine non-Markovianity and coherence, we propagate the system once without excitation for 2 ps and again for 2 ps after excitation, with a Gaussian laser pulse,

$$\begin{aligned} \hat{H}_{\text{laser}}(t) &= -\hat{\mu}\cdot\mathcal{E}(\mathbf{r},t) \\ &= -\hat{\mu}\cdot\left[\chi E(t-\tau_p)e^{-i\omega t + i\mathbf{k}\cdot\mathbf{r}}\right] + \text{c.c}, \end{aligned} \tag{8}$$

where $\hat{\mu} = \mu_{\text{eg}}\hat{S}$, ω is the frequency of the electric field, $\mathcal{E}(\mathbf{r},t)$, with the associated wave vector $\mathbf{k}$ and electric field strength χ. In our simulations, the electric dipole approximation is applied such that, $|\mathbf{k}\cdot\mathbf{r}| \ll 1$. Therefore, a Taylor expansion leads to $e^{i\mathbf{k}\cdot\mathbf{r}} \approx 1$, and the

electric field loses its spatial dependence.[1] The Gaussian field envelope $E(t-\tau_p)$ centred at time τ_p is expressed as:

$$E(t-\tau_p) = \exp\left(\frac{-(t-\tau_p)^2}{2\varsigma^2}\right). \tag{9}$$

The system density matrix was initialized in the ground electronic state, $\rho(t=0) = |g\rangle\langle g|$. To establish sufficient system–bath correlations before excitation, the auxiliary density operators (ADOs) were first propagated for 2.00 ps with a hierarchy truncation parameter of $\xi = 3500$ cm^{-1}. Following this initialization, the system was excited via a semi-classical Gaussian laser pulse centred at $\tau_p = 50$ fs. The total electric field $\mathcal{E}(t)$ is defined by an electric field strength $\chi = 10^{11}$ V m^{-1}, a frequency tuned to the molecular electronic transition ($\omega_{eg} = 16000$ cm^{-1}), and a field envelope with a temporal full-width at half-maximum (FWHM) of 20 fs, chosen to ensure a robust population transfer into the polaritonic manifold. This field strength allows for a high degree of initial distinguishability between the ground and excited states, maximizing the signal-to-noise ratio for the subsequent BLP and l_1-norm measures. The standard deviation ς is related to the Full-Width at Half-Maximum (FWHM) by FWHM $= 2\sqrt{2\ln 2}\varsigma$.

2 Dynamics

To calculate the complex dynamics of our open quantum system, we employ the hierarchical equations of motion (HEOM), to evolve our system through time: this will be done using two different bath regimes. We use Lindbladian jump operators to represent the cavity coupled environment, allowing only for photon loss. We can add this additional interaction with minimal increase to computational cost. These 'environments' are separate, coupling to different parts of the system Hamiltonian. The Lindblad jump operators are implemented as a modification to the existing hierarchy. Similar methodologies have previously incorporated HEOM-Lindblad formalism in the context of polariton dynamics.[2–4] The Lindblad dissipators are added as a modification to the ADO hierarchy, allowing for a secondary dissipative

channel. This approach ensures that Markovian dissipation (cavity loss) and non-Markovian bath correlations are treated with consistency. Since the HEOM is a linear decomposition of the total system-bath dynamics, any additional independent dissipative channel acting on the system degrees of freedom must propagate through the entire hierarchy to maintain the integrity of the system-bath correlations during the dissipative process.

We expand the correlation function as a linear combination of exponential decay terms, capturing the system's dynamics via the prefactors c_k and the bosonic Matsubara frequencies ν_k for $k \in \{0, 1, \ldots, M\}$.

$$C(t) = \sum_{k=0}^{M} c_k e^{-\nu_k t}. \tag{10}$$

Where $\zeta = \sqrt{\omega_0^2 - \frac{\gamma^2}{4}}$,

$$\nu_0 = \frac{\gamma}{2} - i\zeta, \tag{11}$$

$$\nu_1 = \frac{\gamma}{2} + i\zeta, \tag{12}$$

$$\nu_k = \frac{2\pi(k-1)}{\hbar\beta}, \tag{13}$$

$$c_0 = \frac{\hbar\eta\omega_0^2}{2\zeta}\left\{\coth\left(\frac{\hbar\beta}{2}\left(\zeta + i\frac{\gamma}{2}\right)\right) - 1\right\}, \tag{14}$$

$$c_1 = \frac{\hbar\eta\omega_0^2}{2\zeta}\left\{1 - \coth\left(\frac{\hbar\beta}{2}\left(-\zeta + i\frac{\gamma}{2}\right)\right)\right\}, \tag{15}$$

$$c_k = -\frac{4\eta\gamma\omega_0^2}{\hbar\beta}\frac{\nu_k}{(\omega_0^2 + \nu_k^2)^2 - \gamma^2\nu_k^2}. \tag{16}$$

A hierarchy of auxiliary density operators (ADOs) can then be derived and propagated,

given as

$$
\begin{aligned}
\dot{\rho}^{\mathbf{n}}(t) &= -\left(\frac{i}{\hbar}\hat{H}_S^{\times} + \sum_{k=0}^{M} n_k\nu_k - \sum_{k=M}^{\infty}\hat{V}^{\times}\hat{\Psi}_k\right)\rho^{\mathbf{n}}(t) \\
&\quad + \sum_{k=0}^{M}\hat{V}^{\times}\rho^{n_k^+}(t) + n_0\hat{\Theta}^{-}\rho^{n_0^-}(t) + n_1\hat{\Theta}^{+}\rho^{n_1^-}(t) \\
&\quad + \sum_{k=2}^{M} n_k\nu_k\hat{\Psi}_k\rho^{n_k^-}(t) \\
&\quad + \sum_{i}\Upsilon_i\Big(\hat{L}_i\rho^{\mathbf{n}}\hat{L}_i^{\dagger} - \frac{1}{2}\{\hat{L}_i^{\dagger}\hat{L}_i, \rho^{\mathbf{n}}\}\Big), \qquad (17)
\end{aligned}
$$

where

$$
\begin{aligned}
\hat{\Psi}_k &= \frac{4\eta}{\hbar\beta}\frac{\gamma\omega_0^2}{(\omega_0^2+\nu_k^2)^2-\gamma^2\nu_k^2}\hat{V}^{\times}, \qquad (18) \\
\hat{\Theta}^{\pm} &= \frac{\eta\omega_0^2}{2\zeta}\left\{\mp\hat{V}^{\circ} \pm \coth\left(\frac{\hbar\beta}{2}\left(\mp\zeta + i\frac{\gamma}{2}\right)\right)\hat{V}^{\times}\right\}, \qquad (19)
\end{aligned}
$$

and $\hat{V}^{\times}\rho = [\hat{V}, \rho]$, $\hat{V}^{\circ}\rho = \{\hat{V}, \rho\}$ denote the commutator and anti-commutator of the bath coupling, respectively. In the overdamped limit, the solution for the correlation function maintains the same exponential expansion structure as Eq. (10, where

$$
\nu_0 = \Lambda, \qquad (20)
$$

$$
\nu_k = \frac{2\pi k}{\hbar\beta}, \qquad (21)
$$

$$
c_0 = \eta\Lambda\left(\cot\left(\frac{\hbar\beta\Lambda}{2}\right) - i\right), \qquad (22)
$$

$$
c_k = \frac{4\eta\Lambda}{\hbar\beta}\left(\frac{\nu_k}{\nu_k^2-\Lambda^2}\right), \qquad (23)
$$

and the hierarchy of auxiliary density operators (ADOs) can then be derived as

$$\begin{aligned}\dot{\rho}^{\mathbf{n}}(t) =& -\Big(i\hat{H}_M^{\times} + \sum_{k=0}^{M} n_k \nu_k\Big)\rho^{\mathbf{n}}(t) \\ & - \Big(\frac{2\eta}{\hbar\beta\Lambda} - \eta\cot\frac{\hbar\beta\Lambda}{2} - \sum_{k=1}^{M}\frac{c_k}{\nu_k}\Big)\hat{V}^{\times}\hat{V}^{\times}\rho^{\mathbf{n}}(t) \\ & - i\sum_{k=0}^{M}\hat{V}^{\times}\rho^{n_k^{+}}(t) \\ & - i\sum_{k=0}^{M} n_k\Big(c_k\hat{V}\rho^{n_k^{-}}(t) - c_k^{*}\rho^{n_k^{-}}(t)\hat{V}\Big) \\ & + \sum_{i}\Upsilon_i\Big(\hat{L}_i\rho^{\mathbf{n}}\hat{L}_i^{\dagger} - \frac{1}{2}\{\hat{L}_i^{\dagger}\hat{L}_i, \rho^{\mathbf{n}}\}\Big),\end{aligned} \tag{24}$$

where $\rho^{\mathbf{n}}$ is equal to the reduced density matrix if all indices involved in the equation are equal to 0. $\hat{L}_i$ is a collection of jump operators that allow for the dissipation of the system to be calculated. To converge the hierarchy, we use:[5–7]

$$\frac{2M\pi}{\hbar\beta} > \xi, \tag{25}$$

with M as the number of Matsubara frequencies, and the hierarchy depth controlled by

$$\sum_{k=0}^{M} j_k|\mathbf{Re}(\nu_k)| > \xi. \tag{26}$$

The hierarchical equations of motion (HEOM) were propagated for 2 ps at a constant temperature of 298 K. Numerical convergence was ensured by the ξ parameter, with Lindblad dissipators representing photon loss ($\kappa = 0.005$ cm^{-1}) acting across the entire hierarchy of ADOs to maintain consistent system–bath correlations. ξ is chosen to be 10 times larger than the vibrational frequency handled by the HEOM. Therefore, M is sufficient for convergence, satisfying equation 25. The molecular system comprised two electronic states, each coupled to a vibrational mode truncated at $v_{\max} = 3$ states, while the cavity Fock space was truncated

at $N = 3$ states (including the vacuum state). Following the laser pulse, the states were propagated for a further 2 ps to provide the data used for coherence and trace distance calculations.

3 Additional Results

3.1 BLP

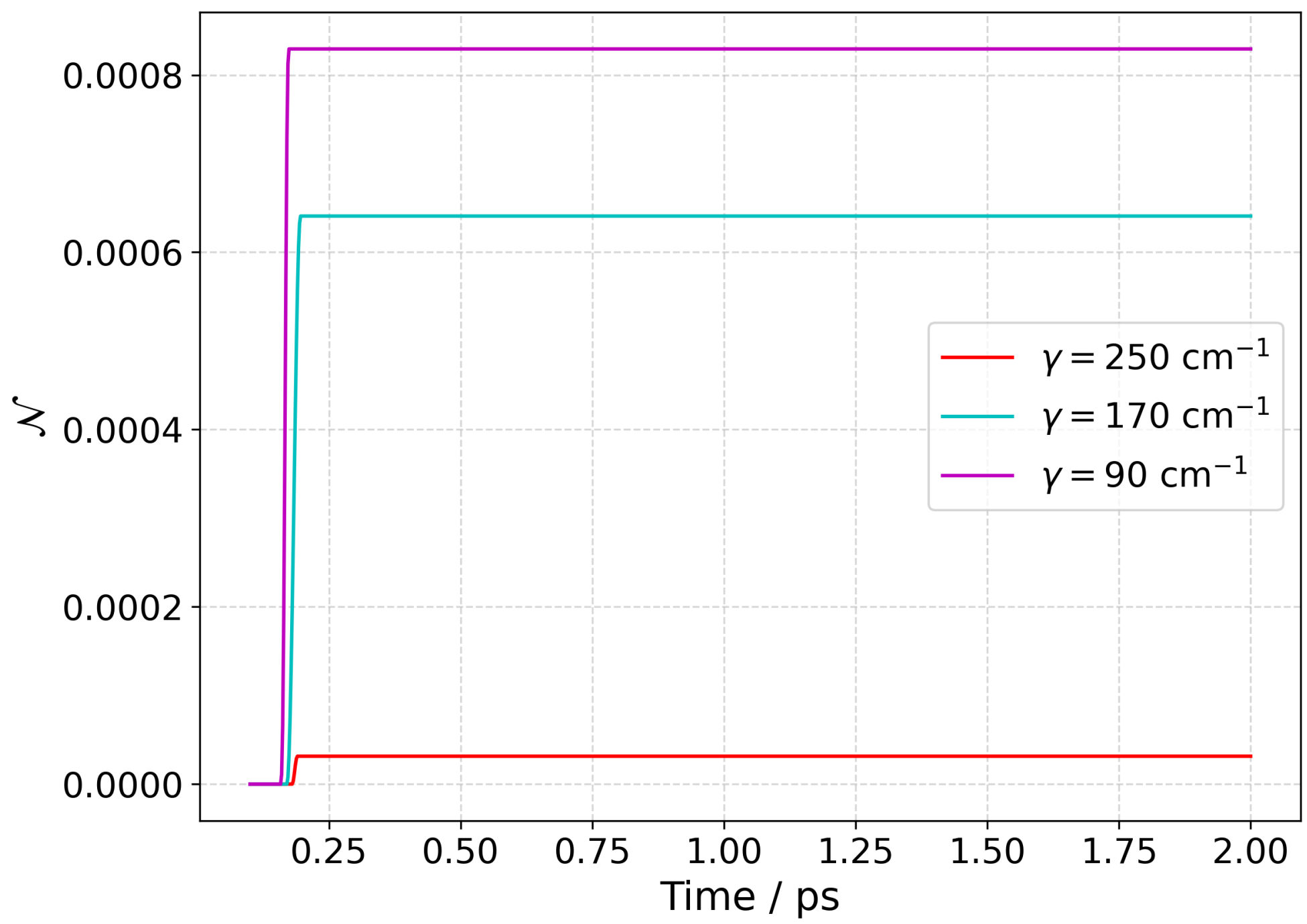

Figure 1: BLP measure for the cavity coupled system, with an underdamped bath, where $\kappa = 0$ for $\gamma = 250, 170$ and 90 cm^{-1}. There is a significant decrease in $\mathcal{N}$ for all values of γ, despite no photon loss from the cavity.

To isolate the influence of light-matter hybridisation from irreversible cavity dissipation, we evaluate the accumulated BLP measure in the lossless limit ($\kappa = 0$). As shown in Figure 1 and Table 1, introducing strong light–matter coupling ($g = 1000$ cm^{-1}) in a lossless cavity systematically reduces the accumulated non-Markovianity relative to the uncoupled baseline across all underdamped bath damping rates (γ). When finite photon leakage ($\kappa > 0$) is reintroduced, the open cavity decay channel provides an irreversible sink for subsystem correlations, reducing the accumulated trace distance derivative to zero. Removing the

Markovian photon loss demonstrates that the observed suppression of environmental memory backflow is an intrinsic feature of polaritonic hybridisation rather than cavity dissipation.

Table 1: Accumulated BLP measure $\mathcal{N}(t = 2$ ps) for underdamped molecular systems across cavity coupling and dissipation regimes.

**Bath Damping**	**No cavity** ($g = 0$ cm^{-1})	**Lossless cavity** ($g = 1000$ cm$^{-1}, \kappa = 0$)	**Leaking cavity** ($g = 1000$ cm$^{-1}, \kappa > 0$)
$\gamma = 250$ cm^{-1}	3.40×10^{-5}	3.10×10^{-5}	0.00
$\gamma = 170$ cm^{-1}	6.45×10^{-4}	6.41×10^{-4}	0.00
$\gamma = 90$ cm^{-1}	4.93×10^{-3}	8.29×10^{-4}	0.00

References

(1) Mukamel, S. *Principles of nonlinear optical spectroscopy*; Oxford university press, 1995.

(2) Ying, W.; Mondal, M. E.; Huo, P. Theory and quantum dynamics simulations of exciton-polariton motional narrowing. *J. Chem. Phys.* **2024**, *161*, 064105.

(3) Fang, J.; Chen, Z.-H.; Su, Y.; Zhu, Z.-F.; Wang, Y.; Xu, R.-X.; Yan, Y. Coherent excitation energy transfer in model photosynthetic reaction center: Effects of non-Markovian quantum environment. *J. Chem. Phys.* **2022**, *157*, 084119.

(4) Huang, Y.-T.; Kuo, P.-C.; Lambert, N.; Cirio, M.; Cross, S.; Yang, S.-L.; Nori, F.; Chen, Y.-N. An efficient Julia framework for hierarchical equations of motion in open quantum systems. *Commun. Phys.* **2023**, *6*, 313.

(5) Dijkstra, A. G.; Prokhorenko, V. I. Simulation of photo-excited adenine in water with a hierarchy of equations of motion approach. *J. Chem. Phys.* **2017**, *147*, 064102.

(6) Chen, L.; Zheng, R.; Shi, Q. . Optical line shapes of molecular aggregates: Hierarchical equations of motion method. *J. Chem. Phys.* **2009**, *131*, 094502.

(7) Green, D.; Humphries, B. S.; Dijkstra, A. G.; Jones, G. A. Quantifying non-Markovianity in underdamped versus overdamped environments and its effect on spectral lineshape. *J. Chem. Phys.* **2019**, *151*, 174112.
